# The Pathophysiology of Cardiac Troponin Release and the Various Circulating Cardiac Troponin Forms—Potential Clinical Implications

**DOI:** 10.3390/jcm14124241

**Published:** 2025-06-14

**Authors:** Johannes Mair

**Affiliations:** Department of Internal Medicine III—Cardiology and Angiology, Medical University of Innsbruck, Anichstrasse 35, A-6020 Innsbruck, Austria; johannes.mair@i-med.ac.at; Tel.: +43-512-504-24118; Fax: +43-512-504-22767

**Keywords:** cardiac troponin, release, pathophysiology, myocardial injury, acute myocardial infarction, circulating troponin forms

## Abstract

Current routine high-sensitivity cardiac troponin assays are the criterion standard for the laboratory diagnosis of myocardial injury due to their high analytical sensitivity and specificity. However, in daily clinical practice, unexpectedly elevated cardiac troponin test results without an obvious clinical correlate are becoming more frequent compared with previous cardiac troponin assay generations. In these patients, myocardial injury may sometimes be undetected by imaging techniques, including cardiac magnetic resonance imaging. This has led to an increased interest in the pathophysiology of cardiac troponin release, particularly with regard to whether troponin can be released in the absence of myocardial necrosis and thereby resulting in an increase in cardiac troponin in the systemic circulation. Although there is in vitro evidence that cardiac biomarkers are released from reversibly injured cultured cardiomyocytes, there is still a lack of evidence for cardiac troponin release apart from different forms of cell death (i.e., apoptosis or necrosis) in animal experiments. Conversely, various circulating cardiac troponin forms have been identified in human blood samples using different analytical methods, raising the question of whether the cause of myocardial injury can be reliably determined by measuring specific circulating cardiac troponin forms. Preliminary clinical data suggests that testing for specific circulating troponin forms could increase the specificity of cardiac troponin for diagnosing acute myocardial infarctions caused by an acute coronary syndrome. This review aims to provide an up-to-date overview of these current cardiac troponin research topics with their potential clinical implications. Typical clinical cases illustrate how to interpret cTn in the individual patient and how to derive a correct diagnosis.

## 1. Background

Since the 1950s, alongside the electrocardiogram (ECG), laboratory parameters for the detection of myocardial injury have been a cornerstone in the clinical assessment of patients with suspected acute myocardial infarction (AMI) [1,2,3]. However, the landscape of cardiac biomarkers has evolved dramatically since then. There has been an ongoing search for more sensitive and cardiac-specific laboratory parameters for the diagnosis of AMI, finally resulting in the development of immunoassays for the detection of cardiac troponins (cTn) [1]. To improve the early diagnosis of AMI, the analytical sensitivity of cTn assays has steadily been increased with each new assay generation, while maintaining their cardiospecificity. This has led to the development of so-called “high-sensitivity” (hs) cTn routine assays, which can detect circulating cTn in the majority of healthy individuals [2]. Thus, for the diagnosis of myocardial injury, cardiac troponin I (cTnI) and cardiac troponin T (cTnT) have become the preferred laboratory parameters, as they are currently the most sensitive and cardiac-specific available for routine laboratories [1,2,3,4,5,6]. However, due to the high analytical sensitivity of current hs-cTn assays, the positive predictive value of hs-cTn for AMI is markedly lower than that of previous cTn assay generations [2,4,5]. Therefore, unexpectedly elevated cTn test results without an obvious clinical correlate are becoming increasingly common and challenging in daily clinical practice [4,5,6]. In most of these patients, these cTn elevations are due to acute or chronic myocardial injury related to various cardiac or non-cardiac pathologies involving the heart, but which are not an AMI [3,4,5,6]. In some of these patients, myocardial injury may even go undetected by imaging due to the higher sensitivity of hs-cTn assays, which can detect cTn in blood samples at femtomolar concentrations [3,4,5,6]. The huge variety of causes of myocardial injury is summarized in Table 1 [1,2,3,4,5,6,7]. These mechanisms include myocardial ischemia caused by an acute coronary syndrome (ACS), which is caused by coronary plaque rupture or erosion with intracoronary thrombus formation (type 1 AMI); myocardial ischemia or hypoxia unrelated to an ACS (type 2 AMI); and myocardial injury unrelated to myocardial ischemia or hypoxia, such as inflammation (e.g., myocarditis), increased myocardial wall stress (e.g., heart failure), toxic injury to the myocardium and trauma (e.g., cardiac contusion) [4,5]. Analytically false-positive test results are very rare [6,8,9].

However, many aspects of cTn degradation within the myocardium, its release, and its subsequent degradation and clearance from the human circulation are still not fully understood. This review aims to provide an update on the current knowledge on cTn degradation, release, and on circulating human cTnI and cTnT forms with their clinical implications. It highlights gaps in current knowledge and the potential future application of measuring different circulating cTn forms for the differential diagnosis of diseases leading to myocardial injury. The problems of cTn interpretation in patients presenting with specific clinical scenarios in daily routine are summarized. Typical clinical cases are presented (mostly as supplemental figures) to illustrate how to interpret cTn in the individual patient and how to derive a correct diagnosis.

## 2. The Pathophysiology of Cardiac Troponin Release from Injured Myocardium

The cTn complex is part of the thin filaments of the myocardium, and its biochemistry and pathophysiology have been reviewed extensively previously [10,11,12]. In summary, the cTn complex plays a central role in the calcium-dependent regulation of actin thin filament function, and it is, therefore, essential for controlling striated muscle contraction. Only two of the three troponins are encoded as cardiac-specific isoforms (cTnI and cTnT) in humans (see Table 2): troponin I (TnI, about 24 kDa), the actomyosin adenosine triphosphatase inhibitory subunit, which confers calcium sensitivity to striated muscle contraction; and troponin T (TnT), the tropomyosin-binding subunit (about 35–36 kDa depending on the additional cTnT isoform variation; the latter is a result of alternative splicing). cTnT and cTnI have a cardiac-specific N-terminal extension. However, troponin C (TnC, about 18 kDa), the calcium-binding subunit, which initiates the sequence of conformational changes on the thin filament, is also expressed in slow-twitch skeletal muscle fibers [13,14,15,16,17].

**Figure 1:**
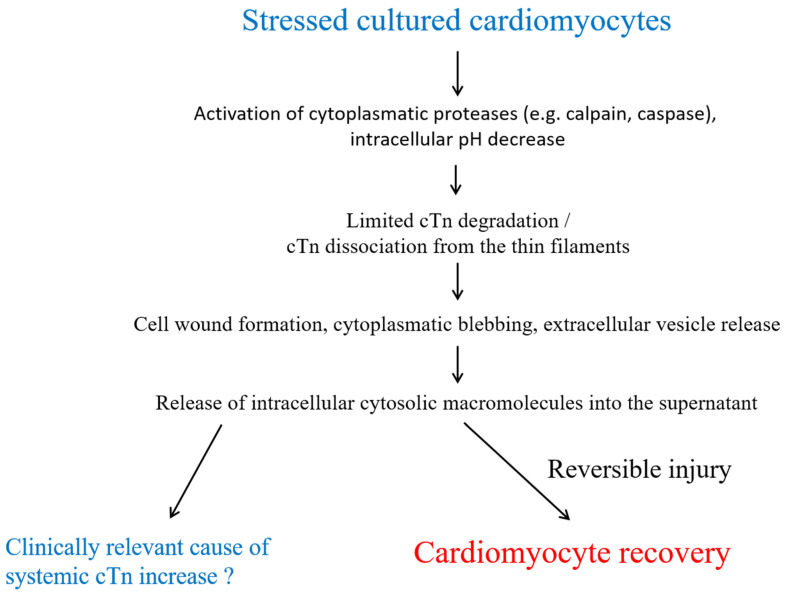
Potential mechanisms of cardiac troponin release from reversibly injured cardiomyocytes. Degraded and dissociated cTn is released into the supernatant via cell wounds, cytoplasmic blebbing, and the formation of extracellular vesicles through a temporarily leaky plasma membrane. Abbreviations: cardiac troponin (cTn).

**Figure 2:**
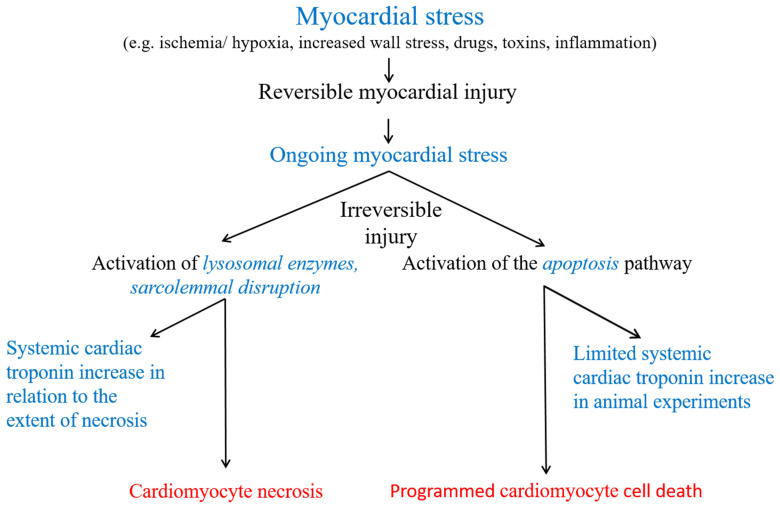
Potential cardiac troponin release mechanisms from injured myocardium. Cardiomyocyte necrosis is the only generally accepted cause of a cTn increase into the systemic circulation in humans. In animal experiments, cardiomyocyte apoptosis was associated with cTn increases into the systemic circulation.

Initially, small “cytosolic” pools of cTnI and cTnT (approximately 5% of the total content) were reported in human myocardium [18,19,20]. However, given the preparation protocols employed by the researchers and the poor solubility of cTnI and cTnT in the hydrophilic sarcoplasma, a more fitting term would be “rapidly degraded, loosely bound, early releasable pool”. When not incorporated into myofilaments, cTnT, for example, is rapidly degraded within cardiomyocytes by enzymes such as caspase or µ-calpain to avoid toxic effects [21,22,23,24]. µ-calpain is activated by increased cytoplasmic calcium, an important and early feature of cell injury [21,22,23,24]. Increased cytoplasmic calcium also activates phospholipases and endonucleases [25]. pH-dependent dissociation of the troponin complex may be another important factor in early cTn release from injured myocardium via a temporarily leaky plasma membrane (see Figure 1) [26]. Subsequent data suggest that the proportion of cTnT that can dissociate rapidly from myofibrils in vivo is substantially higher than the 5–10% previously reported [27]. cTnI is also a substrate of caspase-3, which has a key role in apoptosis; it targets the N-terminal region of cTnT (see Figure 2) [24]. Therefore, all cTn released is likely to be of myofibrillar origin. The pathophysiological background of the limited N-terminal intramyocardial proteolysis of cTn by caspase and calpain may be that cTns are involved in the sensitivity of the myocardium to decreases in intracellular pH, and thus, this may be an early specific functional adaptive response to myocyte injury rather than a simple destructive degradation [21,22,23,24]. Intramyocardial posttranslational modifications of cTn, such as oxidation or phosphorylization, may enhance or inhibit the susceptibility to proteolysis [21,23,24,28,29,30].

Cell death is preceded by a substantial reversible pre-lethal phase (see Figure 1) [26]. However, ongoing myocardial stress, depending on the mechanism and extent of myocardial stress, may lead to different forms of cell death (see Figure 2). When myocardial necrosis occurs, lysosomal enzymes degrade the contractile filaments, and the integrity of the plasmalemma is disrupted. Cardiomyocytes rapidly release intracellular macromolecules into the interstitial space. cTn shows a rapid increase after myocardial injury, comparably fast to cytosolic proteins [19,20,31,32,33,34]. There, additional proteolysis of troponins may occur, e.g., by matrix metalloproteinase-2 or thrombin [35,36]. Notably, however, the currently commercially available routine hs-cTnT assay, which utilizes antibodies targeting the amino acid fragment 125–147, is unaffected by thrombin-mediated cTnT degradation [37]. Local blood and lymphatic flow also influence the onset of the subsequent cTn increase in the systemic circulation. For example, early reperfusion of the infarct-related coronary artery results in more rapid extraction and clearance of cTn from damaged myocardium [38,39,40].

### 2.1. Experimental Data on the Release of cTn from Reversibly Injured Cardiomyocytes or in the Absence of Myocardial Necrosis

There is evidence from in vitro experimental models, that macromolecules, including cTn, are released in the absence of histological evidence of cardiomyocyte necrosis [26,41,42,43,44,45]. For example, cytoplasmic blebbing may already occur during the reversible phase of cell injury [26]. These blebs contain intracellular macromolecules and detach with resealing of the plasma membrane without cell death in cell culture experiments. This may therefore be responsible for limited cTn release from cardiomyocytes [26,41]. Furthermore, it has been reported in cell culture experiments, that tachycardia may stimulate integrins, thereby triggering the release of cTnI in the absence of necrosis [42]. Additionally, the cTn content of cardiomyocytes decreases prior to necrosis [46].

Recent large-animal experiments in pigs by Weil et al. found cTnI increases in peripheral blood samples in the absence of myocardial necrosis [47,48]. cTnI increased after applying left anterior descending artery (LAD) occlusion of only 10 min, which was associated with reversible myocardial dysfunction but without histological evidence of myocardial necrosis in tissue analysis [47]. Apart from ischemia or hypoxia, mechanical stretch in response to pressure or volume overload may trigger the activation of proteases associated with intracellular troponin degradation and the release of troponin fragments from injured cardiomyocytes without evidence of necrosis despite reversible ventricular dysfunction [48]. In this model, an intravenous infusion of phenylepinephrine led to preload-induced myocardial injury by acutely increasing wall tension. While these experiments demonstrate cTn release in response to myocardial ischemia or increased myocardial wall tension in the absence of myocardial necrosis, these results do not prove release from reversibly injured myocardium, as apoptosis, another form of cell death, was detected.

### 2.2. Clinical Data Suggesting the Release of cTn in the Absence of Myocardial Necrosis

Since the publication of the first research radioimmunoassay for cTnI detection [49], the analytical sensitivity of current hs-cTn assays has improved dramatically from 500 ng/L to approximately 1–3 ng/L [37]. It has been postulated that for every 1 µg of human myocardium injured, the cTnI and cTnT concentrations increase by approximately 4 ng/L in peripheral blood samples [50]. This advancement in the analytical performance of cTn assays, while maintaining cardiospecificity, has led to a dramatic improvement in the clinical sensitivity for detecting myocardial injury, that was previously unimaginable at the beginning of clinical cTn research. It is higher than all currently available imaging modalities [51,52].

Initially, cTn could not be detected in healthy individuals; however, with hs-cTn testing, cTn can now be detected in over 50% of healthy individuals [1,2,37,49,53,54]. As analytical interferences have been ruled out [53,54], detectable hs-cTn concentrations in humans with normal hearts suggest a constant limited turnover of cTn and/or cardiomyocytes with cTn being released into the systemic circulation. In a functioning sarcomere, protein synthesis, processing, and degradation occur continuously as a part of physiological turnover [55,56]. cTnT undergoes rapid turnover with a half-life of approximately 3.5 days within cardiomyocytes [56]. If not incorporated into myofilaments, cTnT is rapidly degraded to avoid toxic effects [18,55].

It is probably impossible to prove the release of cTn from reversibly injured myocardium in humans in the absence of different forms of cell death. However, there are increasing numbers of reports in the literature on cTn release in clinical scenarios in which myocardial necrosis is highly improbable [57,58,59,60,61]. For instance, a very sensitive assay demonstrated cTnI release in humans following nuclear perfusion scintigraphy with peak concentrations associated with the extent of myocardial ischemia during stress testing [57]. Furthermore, it has been reported that even normal individuals exhibit elevated circulating cTn levels in response to dobutamine stress or exercise testing [58,59]. Small, but significant cTn concentration increases in coronary sinus blood samples were found within 30 min after brief episodes of incremental rapid atrial pacing using a protocol with comparable myocardial stress to moderate brief physical exercise [60]. This cTnT release in coronary sinus blood was subsequently mirrowed in a delayed, small but significant hs-cTnT concentration increase in peripheral blood samples after 3 h (doubling to tripling of baseline values), even in the subgroups without significant coronary artery disease (CAD) or without net myocardial lactate production proving myocardial ischemia. In all patients without significant CAD and no net myocardial lactate production, hs-cTnT concentrations remained within the upper reference limit (URL).

#### 2.2.1. Clinical Implications: Challenges in Interpreting hs-cTn Concentrations Following Elective Percutaneous Coronary Interventions

**Figure 3:**
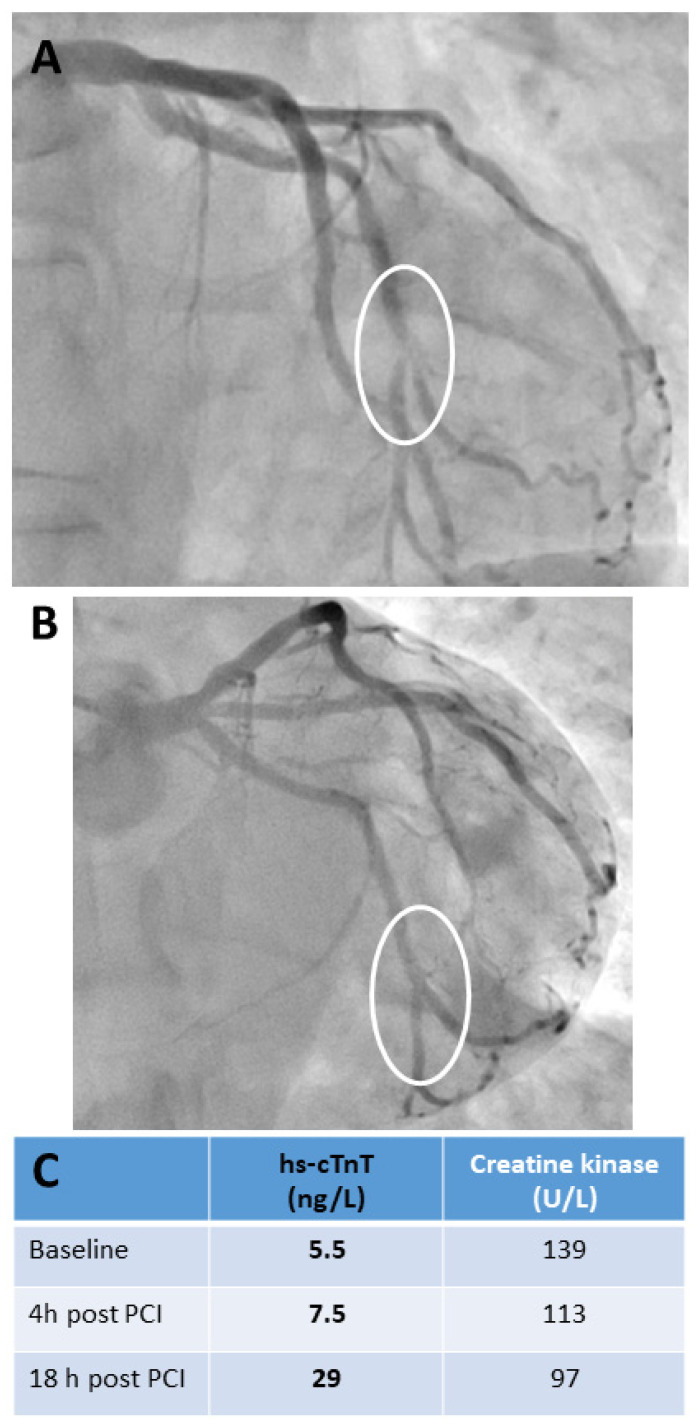
Cardiac biomarker release in a patient undergoing uncomplicated bifurcation percutaneous transluminal coronary intervention for unstable angina. This case clearly demonstrates the importance of specifying exactly when a proposed cTn decision limit should be used to diagnose peri-interventional AMI after elective PCI. A 55-year-old male with a history of CAD was admitted with symptoms of unstable angina. He underwent PCI of the left circumflex coronary artery 11 years ago. Serial ECGs before and after PCI showed no signs of acute myocardial ischemia. Serial hs-cTnT testing in the emergency department before PCI revealed concentrations within the normal range without a significant change. The criteria for AMI were not met. Urgent coronary angiography revealed a subtotal in-stent restenosis at the distal end of the stent involving the distal circumflex artery bifurcation (see (**A**), marked with a circle). There was no other significant stenosis. PCI was performed with an excellent primary result without any complications (see (**B**), marked with circle). The biomarker time courses are listed in (**C**). hs-cTnT showed no significant increase within 4 h after PCI, but increased significantly above the upper reference limit of 14 ng/L the following morning. Creatine kinase activities remained within the reference limit even showing a constant decline; the patient was a tunnel-building construction worker with physically demanding work. Abbreviations: high-sensitivity troponin T (hs-cTnT), percutaneous coronary intervention (PCI), circumflex artery (RCX), coronary artery disease (CAD), acute myocardial infarction (AMI).

cTn increases are found in almost all patients, even those with completely uneventful procedures, after elective percutaneous coronary interventions (PCI) (see Figure 3). This makes it difficult to interpret cTn increases in individual symptomatic patients without knowing their baseline values. When measured with hs-cTn assays, cTn increased significantly in peripheral venous blood samples in individuals with angiographically normal coronary arteries, even after LAD occlusion of only 30 s duration, a setting in which myocardial necrosis is highly unlikely [61]. cTn increases started as early as 15–30 min after balloon occlusion. The highest cTn values were found at the end of the blood sampling period at 4 h after LAD occlusion, with the highest relative increases found after a 90 s lasting LAD occlusion (all participants developed angina, and assay-dependent increases ranged from about 3- to 8-fold baseline values).

Baseline cTn values correlate with the severity and complexity of CAD and may already be increased due to repeated episodes of myocardial ischemia or comorbidities, such as left ventricular hypertrophy or dysfunction. This can lead to increased wall tension in the left ventricle and reduced subendocardial myocardial perfusion (see Supplemental Figure S1) [7,60,62]. In general, the increase in cTn after elective PCI is related to the severity of CAD and the complexity of the interventions performed (see Supplemental Figure S1) [7,62].

Apart from the obvious cases of peri-interventional AMI with complications evident on coronary angiography (see Supplemental Figure S2), its diagnosis is not always straightforward in clinical practice. Applying of the criteria of the 4th Universal Definition of Myocardial Infarction in clinical practice can be challenging, as it requires careful consideration of cTn increase together with clinical symptoms, and evidence for acute myocardial ischemia from angiography, ECG, or imaging [3,7]. However, it has been shown that diagnosing peri-interventional type 4a AMI complicating elective PCI has prognostic implications [63]. It was associated with an about 2-fold higher cardiovascular 1-year event rate and an about 3-fold higher, albeit still low 1-year mortality rate (3%) in patients with normal cTn baseline concentrations [63,64]. Other societies (see Table 3) have opted for a simpler approach based on cTn release, involving markedly higher cTn decision limits in combination with gross ECG evidence for AMI occurrence (e.g., the development of new pathological Q waves) [65,66]. A major limitation of all suggested cTn decision limits is that they do not consider the time-dependence of cTn release after myocardial injury (see Figure 3). No clear criteria are given in all current recommendations on blood sampling regimens or the point in time after PCI at which these decision limits are to be used [3,65,66]. Therefore, all recommendations must be revised to address these issues, although data on the optimal blood sampling regimen after elective PCI is still limited. From a practical point of view, it is suggested that cTn is tested at baseline, which is also useful for risk stratification [7,62,63,64], and, if clinically indicated, 4–6 h after PCI, and in the morning of the next day, if the patient is staying overnight in the hospital. Current clinical practice is usually to only test cTn after PCI when symptoms or complications arise during or after the procedure.

#### 2.2.2. Clinical Implications: Challenges in Interpreting cTn Test Results in Symptomatic Athletes After Competitions or Heavy Endurance Exercise Training Sessions

Even greater debate surrounds the potential clinical significance of cTn increases in asymptomatic endurance athletes (e.g., marathon runners) after competitions or heavy training sessions, with hs-cTn concentrations above the URL and rising and/or falling patterns [59,67,68,69,70,71,72]. Peak concentrations usually do not exceed 3 times the URL, occurring several hours after exercise, with concentrations returning below the URL within 48–72 h [69]. The frequent release of cTn in asymptomatic athletes makes interpreting cTn concentrations in athletes who develop symptoms during or after competitions or training sessions very challenging. A representative clinical case that illustrates the problems encountered in cTn interpreting and deriving the correct diagnosis in symptomatic endurance athletes is presented in Supplemental Figure S3. Depending on the presenting key symptoms, the symptomatic athlete should be assessed as any other high-risk patient admitted to the emergency department. However, the usual post-exercise increase in cTn makes its interpretation more difficult. Serial cTn testing, considering the magnitude of cTn increase and the rate of change, is essential to rule out acute myocardial ischemia as the cause of symptoms, particularly in older (>35 years) male recreational athletes. In the absence of unequivocal ECG findings of acute myocardial ischemia. Performing imaging, in particular coronary computed tomography angiography (CCTA), is also essential.

Elevated cTn concentrations are very common in healthy individuals after extreme endurance exercise [59,68,69,70,71,72]. Following prolonged endurance events, such as marathons, triathlons, ultramarathons, post-race mild and transient acute reversible systolic dysfunction of the right ventricle, but not the left ventricle, has typically been described [69,73,74]. There was no close correlation between post-race cTnI concentrations and decreases in the right ventricular ejection fraction [73]. In marathon runners, the extent of cTn release did not correlate with myocardial injury in imaging [69,73], and the time course of cTn concentrations differed from that of AMI [69,73]. In athletes, post-exercise cTn release is not associated with worse prognosis [69,73,74,75]. However, this appears to be different in physically active elderly individuals from the general population [76]. Experimental data supports the possibility of cTn release from reversibly injured myocardium in athletes. In a rat animal model of an endurance exercise challenge, cTnT release was only associated with reversible changes in cardiomyocyte structure [67]. This finding is corroborated by the observation of minimal cTnT release during and following a marathon in a group of healthy, asymptomatic humans exercising on a motorized treadmill [68]. cTnT increased in all nine participants during running, at completion or within 1 h; subsequently, cTnT returned to baseline values. All but one participant showed a further small increase within a 24 h recovery period, which started several hours after finishing. Five participants still had increased cTnT concentrations 24 h after exercise. The release of cTnT within the first 60 min of running suggests that exercise-induced cTn release is not restricted solely to prolonged endurance exercise. It is highly unlikely that these minor cTn increases reflect myocardial necrosis in these healthy, extremely fit individuals. However, apoptosis cannot be ruled out. A high training volume and long training duration appear to protect against post-exercise cTn release [72]. However, exercise-induced cTn release itself appears to be related to the overall cardiac workload (duration, and in particular, the intensity) of the competition or training session [69]. There was no significant difference in exercise-induced cTn release between patients with a CAD rule-out (calcium score 0 and no plaques in CCTA) and patients with signs of coronary arteriosclerosis in CCTA but without evidence of exercise-induced myocardial ischemia. These individuals were therefore cleared to perform exercise before the study [70]. Nevertheless, despite being clinically asymptomatic, the exercise-induced cTnI release was reported to be higher in individuals with hemodynamically significant CAD [77].

Although cardiac magnetic resonance imaging (MRI) studies of marathon participants found no evidence of myocardial edema or late gadolinium enhancement despite increased cTn concentrations after exercise [69,73,74], its limited sensitivity does not rule out necrosis as the cause of cTn release from the heart. It has been speculated, that long-term exercise training could cause myocardial damage through repetitive high-intensity exercise sessions, which could explain why lifelong endurance athletes exhibit more late gadolinium enhancement compared with their physically less active peers [69,78]. There was also a relation between the extent of late gadolinium enhancement with the number of races competed in and years of training [78].

## 3. Clearance of cTnI and cTnT from the Circulation in Humans

Circulating proteins in the blood can be eliminated in three ways: enzymatic degradation within the circulation; metabolism in organs with a high metabolic rate, such as the liver, pancreas, kidneys, and skeletal muscles; and endocytosis by the reticuloendothelial system. Elimination can also occur via glomerular filtration and excretion in urine. Thus, in cases of impaired clearance from the blood (e.g., renal failure, hypothyroidism) increased concentrations of biomarker may be observed for longer than usual, and baseline values may be higher than normal.

Most proteins released from injured myocardium, including cTn, appear to be catabolized in tissues with a high metabolic rate, particularly in the liver, probably via scavenger receptor-mediated endocytosis and subsequent degradation in lysosomes [79]. In this respect, the reports of cTnT degradation by thrombin [36], a rather indiscriminate serine protease, which cleaves cTnT between R68 and S69, add interesting information and may explain the heparin interference of a previous generation of cTnT assays [36,80]. However, the role of thrombin in cTnT elimination remains to be shown. Small molecules, such as myoglobin, pass through the glomerular filtration membranes of the kidneys and can be found in the urine [81]. These proteins are mainly reabsorbed and subsequently metabolized in the tubular epithelial cells. Thus, prolonged increases in such biomarkers are found in the presence of impaired renal clearance from blood. Intact free cTnT is too large to be filtered by the glomerula, but smaller cTnT fragments are small enough to be cleared by the kidneys. By contrast, cTnI appears to be mainly found in the blood as part of complexes (see below) which are too large for glomerular filtration. At high cTnT concentrations (e.g., after AMI), extra-renal clearance of cTnT dominates [79], but at low concentrations (<100 ng/L), such as in patients with chronic cardiac diseases, renal clearance appears to dominate, as demonstrated in experiments where renal perfusion was reduced [82]. Recent data suggest that, as cTn drops to lower concentrations such as those often observed in patients with stable cTn elevations, cTnT clearance becomes slower and more renal-dependent [82]. Smaller, circulating, degraded cTn fragments, which are predominantly found in the blood in the case of chronic myocardial injury (see below), are small enough for glomerular filtration [82]. However, local degradation and tubular reabsorption could prevent the detection of cTnT and cTnI in urine. At these low steady-state concentrations, cTnT levels are roughly twice as high if kidney function is reduced by 50% [82]. Nevertheless, other mechanisms such as increased myocardial stress and wall tension related to various causes may also contribute to the cTnT elevations observed in patients with chronic renal failure [6,8].

When cTn enters the bloodstream, it follows an exponential two-phase model comprising a distribution and an elimination phase. Once cTnT and cTnI enter the circulation, they are initially cleared with a short half-life of around 0.5 h in dogs and rats [83]. A recent, carefully designed clinical study [84] involving autologous re-transfusion of plasma, which was obtained by plasmapheresis during the subacute phase of AMI, several weeks later, found that the median half-life of cTnT calculated in humans was about 2 h with a clearance of about 80 mL/min. The half-life of cTnI was longer, at about 4 h, with a clearance rate of about 40 mL/min. This study design avoids the problem of ongoing release of cTn from damaged myocardium. However, different cTn fragments may have different half-lives [82,85]. Recently, the half-life of high-molecular-mass (long-form) cTnT was reported to be approximately 9 h as calculated from the analyis of concentration time courses in AMI patients [85]. However, this approach may overestimate the true half-life due to ongoing cTn release from damaged myocardium.

## 4. Circulating Forms of Cardiac Troponin in Human Blood

The biochemical and pathophysiological background includes post-translational modifications, such as phosphorylation and oxidation, intramyocardial N-terminal proteolysis of cTnI and cTnT by caspase and calpain, and the dissociation of the cTn subunits from the cTn complexes, resulting in cytoplasmatic cTn forms, that can be released in response to myocardial injury (see above [10,11,12,28,29,30,45,46,47,48]). Released cTn may undergo further degradation in the interstitial space and the blood [35,36]. Serum or plasma is a complex matrix containing many abundant proteins, such as albumin, and comparably low concentrations of cTn. This presents a significant analytical challenge when trying to characterize circulating cTn forms using standard proteomics techniques, such as mass spectrometry [86,87,88], the criterion standard in proteomics, or gel filtration, as highly sensitive methods are required. In principal, there are two primary approaches: antibody-based techniques and physical separation techniques, such as Western blotting, gel filtration chromatography, and mass spectrometry. All these techniques have their own strengths and limitations (e.g., denaturing conditions and analytical sensitivity). For example, specific sample preparation and purification procedures are required to eliminate this background noise caused by abundant blood proteins to enrich cTn in the sample for detection by mass spectrometry [86,87]. Nevertheless, its analytical sensitivity remains limited, requiring very high cTn concentrations in samples [87]. Denaturing techniques are not suitable for distinguishing between the different forms and sizes of the ternary cTnTIC or cTnIC complexes.

A more straightforward approach would be to develop specific and sensitive immunoassays for detecting particular circulating cTn forms [85], once the relevant circulating cTn forms released in response to different causes of myocardial injury have been characterized using more sophisticated analytical techniques. For practical reasons, therefore, sensitive and specific sandwich immunoassays are the preferred method for research and potential future routine use [85,89,90]. These assays have the potential to be adopted for routine diagnosis on automated, high-throughput platforms.

Table 4 summarizes the currently available data on circulating cTn forms. These data are based on different analytical approaches, mainly Western blotting, gel filtration chromatography, and sandwich immunoassays, each with their own method-specific limitations. The data are also mainly based on analysis of stored samples. Testing of stored samples has limitations compared to testing of freshly drawn blood samples as storage conditions and freeze–thaw cycles may affect in vitro stability and consequently the circulating degradation forms within samples. Additionally, thrombin generation in serum samples may affect in particular cTnT forms. In EDTA plasma samples, the anticoagulant may affect cTn complexes [91], and ideally, the reported results on cTn complexes should be confirmed by testing fresh heparinized plasma samples.

The published results [92,93,94,95,96,97,98,99,100,101,102,103,104,105,106,107,108,109] can be summarized as follows: intact cTn forms disappear rapidly from the circulation in the hours immediately following AMI. After AMI, mainly truncated binary cTnIC with varying quantities of mostly truncated ternary cTnICT complexes are found [92,93,94,96,97]. So-called “large size” cTnIC and cTnTIC complexes are only found early after AMI. Between 75 and 100% of the total cTnI content in the serum of post-AMI patients is part of a complex [92,93,94,100,102,106]. By contrast, cTnT also circulates in significant amounts as a free unbound form [101,103,104]. Intact cTnT is rapidly degraded at the N-terminal end. Early after AMI, it has mainly been described as a high-molecular-mass (HMM, ≥29 kDa), “long” form, including complex-bound cTnT [97,104,105]. Over time, the amount of heavily truncated cTnT forms (14–16 kDa) increases, probably due to degradation at both the C-terminal and N-terminal ends [101,103,104]. In AMI patients, cTnI primarily exists as truncated cTnIC and truncated cTnTIC complexes. The data on cTnI degradation is less consistent, but degradation at both ends has been reported as well [102,106,107]. Unlike the N- and C-terminal regions, the central portions of cTnI and cTnT appear to be stable [94,97,102,104,109]. These epitopes are the best targets for developing a routine cTn immunoassay with high analytical sensitivity and high in vitro stability [37].

Interestingly, the cause of myocardial injury may affect the circulating cTn forms (see Table 4). In the acute phase of AMI, HMM (long) cTnT forms predominate, whereas in chronic end-stage renal disease or in individuals who have performed strenous endurance exercise (e.g., marathon running or triathlons), only LMM cTnT forms could be detected [101,104,105]. These observations suggest that the detection of cTn composition in blood samples could be used in the future to define the cause of myocardial injury and to increase the specificity of cTn testing for AMI.

### 4.1. Commercially Available Routine hs-cTn Assays Aim to Measure Total cTnI or cTnT 

The practical relevance of complex formation, post-translational modifications of cTn and cTn degradation is that these processes may alter the availability of specific epitopes, resulting in different cTn variant recoveries of various cTn assays. The antibodies in currently commercially available hs-cTn assays target epitopes within the stable central regions of the cTnI and cTnT molecules [37]. Consequently, most of these assays detect total cTnI or cTnT, including intact cTn (free or complex-bound), as well as a comprehensive but varying mixture of cTn degradation products [37].

### 4.2. Research cTn Assays That Detect Specific Forms of Circulating cTn

Recently, immunoassays have been developed to quantify intact or only moderately degraded cTnT (referred to as the “long-form”) [85,89,90], which do not detect heavily degraded cTnT, referred to as the “small-form”. In pilot studies, the ratio of “long-form” to total cTnT has shown potential for discriminating acute from chronic myocardial injury, as well as between different etiologies of myocardial injury [90,105,110,111,112,113,114]. Similar findings have not yet been reported for cTnI [106]. Nevertheless, a hs-cTnI/hs-cTnT ratio in blood samples could exhibit comparable discriminatory capabilities for distinguishing type 1 AMI from other causes of myocardial injury [115,116]. The promising results of these pilot studies of testing cTn composition, however, must be confirmed in large-scale clinical studies using fresh blood samples.

## 5. Are There Any Clinically Relevant Differences Between cTnI and cTnT?

When critically reviewing the published literature on this topic, it must be acknowledged, that cTnI and cTnT assays are neither standardized nor harmonized, meaning absolute values are difficult to compare. The only way to make meaningful comparisons is to transform absolute values into multiples of the URL of each assay. However, even then, there may still be a bias if one cTn assay has a markedly higher analytical sensitivity than the other. Therefore, URLs should be used that were calculated from the same reference population (such as the American Association of Clinical Chemistry and Laboratory Medicine reference population biobank) using the same statistical method for URL calculation. In addition, comparisons were primarily based on stored samples, so differences in the in vitro stabilities of cTns must be ruled out as a reason for the observed discrepancies. In many studies, samples were stored for months or even years before measurement. Therefore, further information is often required, such as immediate cTn measurements without sample storage, and studies using cardiac MRI imaging, which is the most sensitive imaging modality currently available for assessing myocardial injury, to confirm the reported differences in the clinical sensitivities and specificities of cTnI and cTnT in specific clinical scenarios. Examples include patients with chronic skeletal muscle myopathies or patients with severe chronic renal failure.

### 5.1. Time Courses of cTnT and cTnI Concentrations After an AMI

Although cTnT has a broader diagnostic window after AMI than cTnI [31,39,40,117], both are considered equally effective in terms of diagnostic performance during the acute phase of AMI [40,118,119]. No clinically relevant differences in their early diagnostic sensitivity for AMI have yet been published. Both cTns generally detect myocardial damage equally well [120].

Following an AMI with occluded coronary arteries, the cTn time courses are influenced by whether or not early tissue reperfusion of the infarcted myocardium has occurred [38,39,40]. Following early reperfusion of the infarct-related coronary artery, cTnT and cTnI peaks’ concentrations are typically found around 12 h after the onset of symptoms (see Supplemental Figure S4). Both troponins exhibit a biphasic release pattern, with a second peak value several days after AMI. However, this feature is much more pronounced in cTnT concentration time courses (see Supplemental Figure S4), with cTnT increases typically lasting a couple of days longer than cTnI increases [40]. These second cTnI peaks are much lower than the initial peaks (see Supplemental Figure S5) and may be overlooked if blood samples are taken infrequently (e.g., only daily) [40]. In AMI patients with ST-elevations (STEMI) who underwent successful primary PCI, they were even absent [117]. If early tissue reperfusion of the infarcted myocardium fails, cTnI peaks occur around 24 h after symptom onset, with the maximum of cTnT concentrations found several days after AMI (see Supplemental Figure S6) [38,39,40]. The cTn concentration time courses of AMI patients without ST-segment elevation (non-STEMI) resemble those in STEMI patients with successful primary PCI [121]. cTn peaks and release correlate with infarct size [33,122,123,124]. Early cTn concentration time courses do not differ significantly between STEMI and non-STEMI patients. Admission concentrations and the rate of increase within the first 1–2 h—although usually higher in STEMI—do not permit a reliable discrimination [120,121]. Similarly, they do not allow an accurate discrimination between AMI patients and patients with myocardial injury unrelated to myocardial ischemia [120].

Despite the lack of differences in early diagnostic sensitivity for AMI [40,118,119], it has been hypothesized that cTnI is released more quickly following myocardial damage [125]. However, as mentioned above, although absolute concentrations of hs-cTnI may be markedly higher, this conclusion is not possible, as the observed differences in absolute values in in vitro and animal experiments may simply be related to differences of cTn assay cross-reactivities with cTn of animals using cTn assays, given that these assays were designed to detect human cTn. The freeze–thawed human myocardial tissue damage model used in one study [125] is highly unphysiological. From a pathophysiological point of view, it is likely that cTnI and cTnT are released from the thin filaments equimolarly in equal amounts after myocardial damage. However, theoretically, cTnT could re-bind to a larger extent to insoluble structures of cardiomyocytes, which remains to be demonstrated.

### 5.2. cTn Discrepancies in Patients with Chronic Skeletal Muscle Diseases

One potential explanation for the commonly reported increased cTnT and normal cTnI concentrations in patients with chronically stressed skeletal muscle [126,127,128,129,130,131,132,133] is that, during fetal development, cTnT is expressed in cardiac and embryonic and neonatal skeletal muscles [16,134,135,136,137]. A representative patient with Becker’s muscular dystrophy is presented and discussed in Supplemental Figure S7. cTnT is down-regulated in skeletal muscle during development and gradually disappears after birth. Healthy adult human skeletal muscle does not contain cTnT. In contrast, cTnI has not yet been reported to be expressed outside the heart so far [10,15]. Chronic injury to human skeletal muscle, as experienced by patients with chronic skeletal muscle myopathies, recapitulates embryonic myogenesis with the re-expression of fetal proteins, which may include cTnT isoforms [134]. The re-expression of these proteins is likely to be dependent on the severity of the disease. These re-expressed proteins can be released into the circulation from damaged skeletal muscles, as occurs in patients with chronic myositis, myopathies, or storage diseases.

The presence of messenger ribonucleic acid (mRNA) coding for cTnT in biopsies from patients with chronic skeletal muscle diseases has been repeatedly reported in the literature [128,130,132,133,138,139,140,141]. Data on the re-expression of cTnT in humans at the protein level in humans are primarily derived from immunohistochemistry and Western blotting [128,133,138,139]. However, given the inherent problems of specificity of these technologies, these observations are not a definitive proof of re-expression. More recently, cTnT expression in skeletal muscle biopsy specimens from patients with Pompe disease was demonstrated by the detection of cTnT fragments using nanoflow LC-MS mass spectrometry, alongside the simultaneous detection of cTnT mRNA [130]. In contrast, cTnT was not detected in the skeletal muscle of healthy controls. Wens et al. [130] detected the cTnT isoform 6, which is the cTnT isoform expressed in healthy hearts, in the skeletal muscle of some patients with Pompe disease. This suggests that skeletal muscle could be a source of circulating cTnT in these patients if cardiac involvement is ruled out. However, Schmid et al. [131] found no evidence of cTnT re-expression in skeletal muscle biopsies from patients with myopathies and myositis using mass spectrometry. Nevertheless, significantly more patients had elevated peripheral blood cTnT concentrations compared to cTnI (see also Supplemental Figure S7). These conflicting results may be explained by differences in cTnT concentrations within skeletal muscle dependent on diseases, and/or differences in the analytical sensitivities of mass spectrometry-based analytical methods used in both studies. The study of Schmid et al. [131] did not test for cTnT mRNA expression in skeletal muscle specimens.

In summary, accumulating evidence suggests that cTnT is re-expressed in several chronic human skeletal muscle diseases. cTnI is the most cardiac-specific marker in these rare patient populations. Although it is easier to interpret when AMI is suspected in these patients, serial testing is still required to confirm acute myocardial injury by a significant change in cTnI concentrations. Furthermore, the frequency of cardiac involvement increases with the age of patients with skeletal muscle myopathies, which may lead to chronic increases in both cTnT and cTnI concentrations.

### 5.3. cTnI and cTnT in Patients with Chronic Renal Failure

As already discussed in above, discrepancies in cTnI and cTnT concentrations are common in patients with advanced renal failure [142,143,144]. This may be due, at least in part, to differences in the renal clearance of cTn fragments from the blood [79,82]. In hemodialysis patients, changes in cTns during hemodialysis depend on the membrane used and the dialysis modality [145,146]. Both influence the permeabilities and clearances of proteins including cTn fragments. In addition, cTn complex and cTn fragment adherence to the dialyzer membrane may differ and be membrane-dependent. The recovery of cTn fragments is assay-dependent as well. Furthermore, myocardial stress due to various causes (e.g., CAD, hypertension, left ventricular hypertrophy, and heart failure) in this patient population may lead to a stable, chronic increase in both cTnI and cTnT. However, cTnI increases are less frequent [142,143,144]. Ricchiuti et al. [140] reported cTnT mRNA expression and cTnT protein expression by Western blot analysis, with no cTnI expression in approximately 50% of skeletal muscle specimens taken from the abdominal wall, back muscles, and arm muscles of hemodialysis patients. It was not reported which muscle specimens tested positive. In contrast, Haller et al. [147] reported the absence of cTnT expression in the skeletal muscle biopsies taken from abdominal wall or back skeletal muscles of five patients with end-stage renal failure. However, truncal skeletal muscles are not usually affected by uremic skeletal myopathy. Despite cTnT increases being more frequently observed and irrespective of the mechanisms of increase, both hs-cTnT and hs-cTnI maintain their prognostic value in patients with chronic renal failure [142]. As with chronic skeletal muscle diseases, cTnI appears to be the most cardiac-specific biomarker for the detection of AMI in this patient population, although serial testing of cTnI is also required to document a significant change in cTnI concentrations.

## 6. Summary and Conclusions

This systematic literature review aims to assist with the routine interpretation of cTn and to provide insight into potential release mechanisms and future developments of cTn testing. It offers practical guidance on cTn interpretation and describes how to derive a correct diagnosis in the individual patient by including discussion of routine clinical cases. The key messages of this review can be summarized as follows:

1. Currently, cTnI and cTnT remain the most accurate laboratory parameters for the routine laboratory diagnosis of myocardial injury, and they will be difficult to replace.

2. An unexpectedly elevated cTn test result should not be ignored, as analytical interferences resulting in false-positive test results are very rare.

3. cTn degradation in response to myocardial injury starts within cardiomyocytes, and cTnI and cTnT are rapidly released after myocardial injury, at a comparable rate to cytosolic proteins.

3. Even very mild forms of myocardial injury, such as those resulting from brief periods of myocardial ischemia or intense myocardial workload, can be reliably detected using hs-cTn assays.

4. Currently, the only generally accepted mechanism of cTn release into the systemic circulation in humans is myocardial necrosis.

5. Other potential mechanisms demonstrated in cultured cardiomyocytes (reversible cell injury) or in animal models (apoptosis, another form of cell death) are very difficult, if not impossible, to prove in humans in the absence of histological tissue analysis.

6. In general, there are no clinically significant differences in the diagnostic performance of cTnI and cTnT. However, after AMI, cTnT tends to remain elevated for a longer period of time than cTnI.

7. In specific, albeit rare, patient populations with chronic skeletal muscle diseases or chronic renal failure, cTnI has a higher clinical specificity than cTnT for the detection of acute myocardial injury.

8. The extreme analytical sensitivity of hs-cTn assays comes at the cost of clinical specificity for AMI.

9. One promising approach to improving the specificity for Type 1 AMI is to measure specific cTn degradation forms (e.g., the so-called “long-form” of cTnT or calculating a cTnI/cTnT ratio). However, these initial results must be confirmed in larger, unselected patient populations.

## Figures and Tables

**Table 1:** Differential diagnosis of cardiac troponin increases in peripheral blood samples: cardiac and non-cardiac diseases leading to myocardial injury.

1. Type 1 AMI
Acute myocardial ischemia caused by coronary atheriosclerotic plaque rupture or erosion with intracoronary thrombus formation (acute coronary syndrome).
2. Type 2 AMI
Acute myocardial ischemia/hypoxia unrelated to an acute coronary syndrome or myocardial hypoxia caused by cardiac oxygen supply/demand imbalance. Coronary non-atherothrombotic cause: embolism, spasm, spontaneous dissection, vasculitis (e.g., Kawasaki disease or Churg–Strauss syndrome). Coronary microvascular dysfunction. Prolonged tachy- or bradyarrhythmias *. Other acute cardiac and pulmonary diseases: e.g., heart failure, pulmonary embolism. Systemic diseases with acute or chronic ischemia or hypoxia *: e.g., prolonged hypo- (e.g., hemorrhagic shock) or hypertension (hypertensive urgency or crisis), acute or severe chronic respiratory failure, acute or severe chronic anemia.
3. Other and partly multifactorial causes of myocardial injury
Inflammation: e.g., acute and chronic myocarditis. Toxic cardiac injury: e.g., drugs, chemotherapeutic agents (e.g., anthacycline, trastuzumab). Trauma: e.g., cardiac contusion, cardiac surgery, cardiac ablation therapy, frequent repeated defibrillator shocks. Myocardial infiltration: e.g., cardiac amyloidosis, cardiac sarcoidosis. Chronic structural heart diseases *: e.g., cardiomyopathies, severe valvular heart diseases. Massive central nervous sympathicus activation *: e.g., with severe cerebral hemorrhage, ischemic stroke, or traumatic brain injury, Takotsubo cardiomyopthy. Multifactorial or unknown causes: e.g., long-lasting or intensive endurance exercise (e.g., after marathon running), sepsis *, chronic severe renal failure.

* Particularly in presence of concomitant coronary artery disease as a bystander disease. Serial cardiac troponin testing is essential for differentiating between acute and chronic myocardial injury by detecting significant changes (>20% from increased baseline values). Abbreviation: acute myocardial infarction (AMI).

**Table 2:** The biochemistry of cardiac troponin isoforms in humans.

Isoform	Gene, Location	Expression	Aminoacids	Molecular Mass
**cTnC**	TNNC1, 3p21.1	Heart, slow-twitch skeletal muscle	161	18.4 kDa
**cTnI**	TNNI3, 19q13.42	Heart	210	24 kDa
**cTnT**	TNNT2, 1q32.1	Heart, skeletal muscle (fetal period, chronic injury)	287–298 *	about 34–36 kDa

See references [13,14,15,16,17]. * There are several cTnT isoforms produced by alternative splicing; the major isoform in normal human hearts is isoform 6 (cTnT3), 34.6 kDa [17]. Abbreviations: cardiac troponin C (cTnC), cardiac troponin I (cTnI), cardiac troponin T (cTnT), kilo Dalton (kDa).

**Table 3:** Suggested criteria for diagnosing peri-procedural AMI in elective PCI.

	cTn Baseline ≤ URL	cTn Baseline > URL	Clinical Criteria (≥1 Needed)
**UDMI** Type 4a AMI [3]	>5× URL plus ECG or imaging criteria	Increase >20% + >5× URL plus ECG or imaging criteria	New signs of myocardial ischemia as evidenced by ECG, imaging, or coronary flow-limiting complications
**SCAI** clinically relevant AMI [65]	≥70× URL or ≥35× URL plus ECG criteria	≥70× URL or ≥35× URL plus ECG criteria	New Q waves in ≥2 contiguous leads New persistent LBBB
**ARC-2** peri-procedural AMI [66]	≥35× URL plus ECG or imaging criteria	≥35× URL plus ECG or imaging criteria	New Q waves or equivalents, evidence in imaging, coronary flow-limiting complications

Abbreviations: percutaneous coronary intervention (PCI), Universal Definition of Myocardial Infarction (UDMI), Society for Cardiovascular Angiography and Interventions (SCAI), Academic Research Consortium (ARC), acute myocardial infarction (AMI), upper reference limit (URL), left bundle branch block (LBBB), cardiac troponin (cTn), electrocardiogram (ECG).

**Table 4:** A summary of the current knowledge regarding circulating cardiac troponin complexes and cardiac troponin forms in relation to the cause of myocardial injury.

	Intact and Truncated cTnTIC Complexes	Free Partially Truncated (HMM) cTnT	Intact and Truncated cTnIC Complexes	Free Heavily Truncated (LMM) cTnT
AMI	x	x #	x *	x
ESRD			x	x
Post heavy endurance exercise (e.g., marathon)			x	x

* The main form of circulating cTnI found in the acute phase of AMI. # The main form of circulating cTnT after AMI (acute phase). According to preliminary clinical results, HMM cTnT is primarily only found in the blood of AMI patients during the acute phase. In contrast, cTnI is mainly found in blood as a partially degraded cTnIC complex in response to all etiologies of myocardial damage. Some is found as a partially degraded cTnTIC complex (e.g., early after AMI), and small amounts are found as free degraded cTnI. Abbreviations: acute myocardial infarction (AMI), end-stage renal disease (ESRD), low-molecular mass (LMM), high-molecular mass (HMM), cardiac troponin I (cTnI), cardiac troponin T (cTnT).

## Data Availability

Data are contained within the article.

## Supplementary Materials

The following supporting information can be downloaded at: https://www.mdpi.com/article/10.3390/jcm14124241/s1, Figure S1: Peri-interventional cTnT interpretation in a 60-year-old male with uncomplicated chronic total occlusion (CTO)-PCI; Figure S2: A patient with angiographically documented peri-interventional type 4a AMI; Figure S3: A symptomatic marathon cyclist; Figure S4: Time course of cTnT concentrations in a patient with successful primary PCI; Figure S5: Time courses of cardiac biomarkers in a 45-year old male with STEMI; Figure S6: Time course of cTnT concentrations in a patient with STEMI and no reflow after primary PCI; Figure S7: cTnI, cTnT, and creatine kinase in an asymptomatic young male with Becker’s dystrophy.
