# Supplementary material for: The Pathophysiology of Cardiac Troponin Release and the Various Circulating Cardiac Troponin Forms—Potential Clinical Implications
Source: J Clin Med. 2025 Jun 14;14(12):4241. doi: 10.3390/jcm14124241 (PMC12194301; doi:10.3390/jcm14124241)
Supplement: Supplementary file 1 [file jcm-14-04241-s001.zip › jcm-3637595-supplementary.pdf]

Supplemental figure S1: Interpretation of peri-interventional cardiac troponin T in a 60-year-old male with known coronary artery disease, who underwent successful, uncomplicated chronic total occlusion (CTO) percutaneous coronary intervention (PCI) of the right coronary artery for stable angina

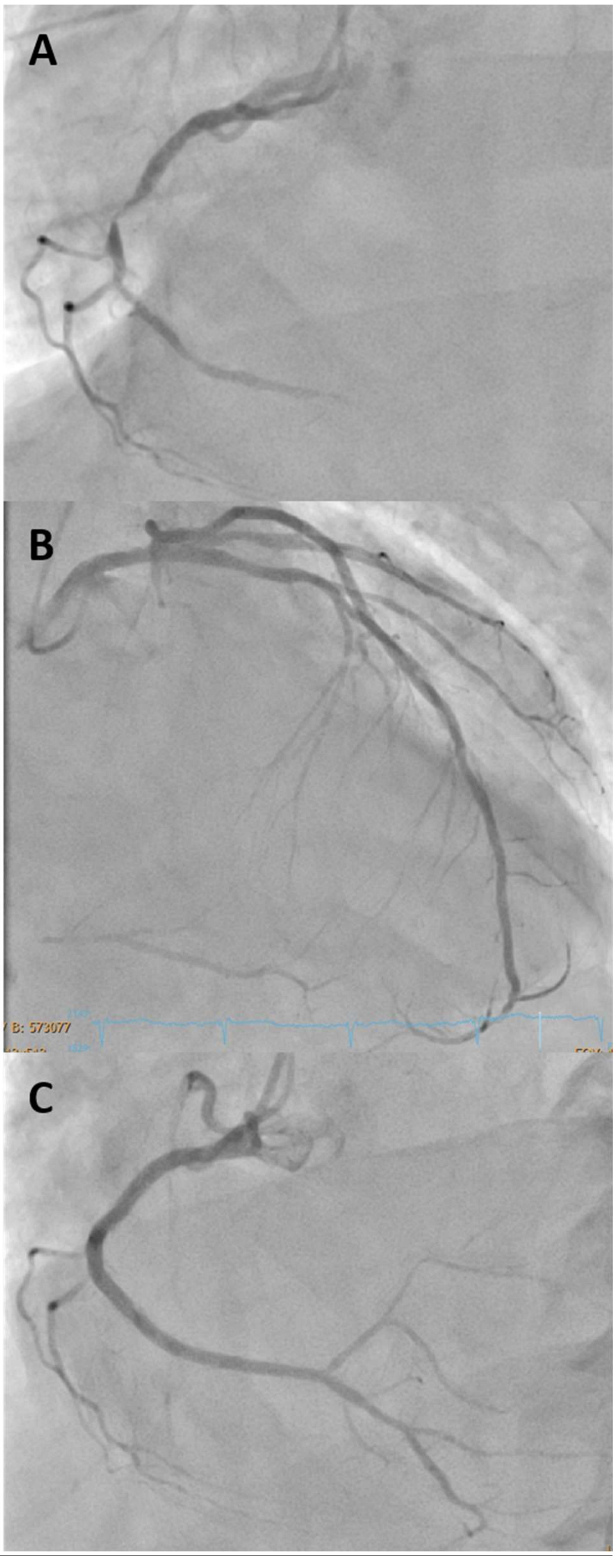

Figure legend S1:

Angiography of the right coronary artery (RCA) before PCI (figure S1 A) shows high-grade stenoses and chronic total occlusion (CTO) at the the crux cordis. Angiography of the left coronary artery (figure S1 B) shows the successful outcome of left main PCI, performed approximately 20 months previously, with collateral flow to the distal RCA. The excellent primary result of the RCA CTO-PCI is shown in figure S1 C.

His baseline hs-cTnT concentration before PCI was 21 ng/l. Approximately 12 hours after CTO-PCI, despite an uneventful course without clinical or ECG signs of peri-interventional myocardial ischaemia, cTnT increased to 117 ng/L. Therefore, despite the concentration being >5-times the upper reference limit (70 ng/L), the criteria for a Type 4a myocardial infarction were not met. There was no significant change in creatine kinase (from 118 to 106 U/L). This patient already had a baseline hs-cTnT concentration above the URL (14 ng/L), indicating chronic myocardial injury. This value was stable; during a hospital stay approximately 2 month earlier in another hospital, it was also 21 ng/L. The causes of chronic myocardial injury in this patient are probably multifactorial. Firstly, he had significant coronary artery disease, presenting with a symptomatic collateralised CTO of the RCA, which led to repeated episodes of myocardial ischemia. Secondly, he had a history of long-term arterial hypertension, presenting with left ventricular hypertrophy and dysfunction (the left ventricular ejection fraction was moderately reduced [33%], and the N-terminal pro B-type natriuretic peptide concentration was markedly increased [3182 ng/L]). These factors both lead to increased wall tension in the left ventricle and reduced subendocardial myocardial perfusion.

Supplemental figure S2: A patient with angiographically documented peri-interventional type 4a acute myocardial infarction

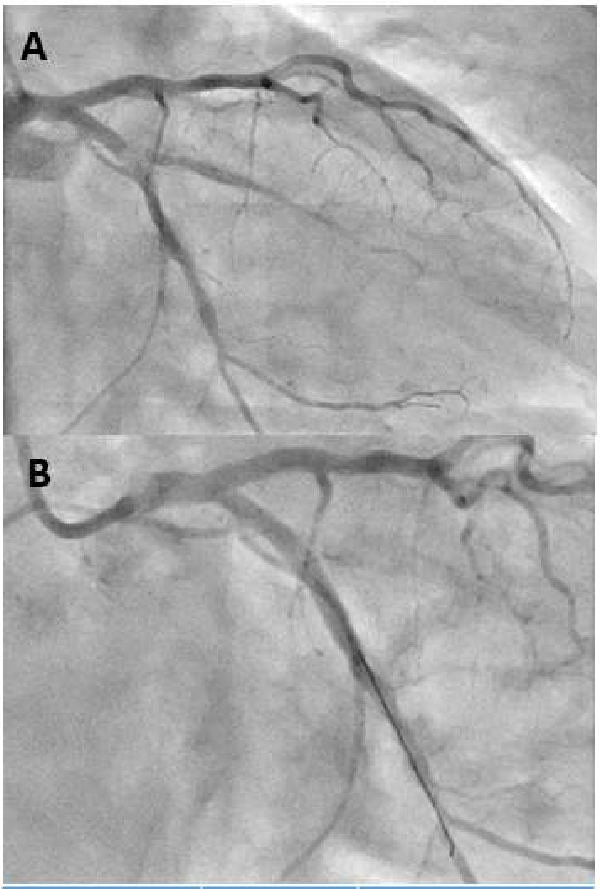

C	hs-cTnT (ng/L)	Creatine kinase (U/L)
Baseline	31.6	68
4 h after PCI	133	86
9 h after PCI	158	99
18 h after PCI	170	117
42 h after PCI	165	93

Figure legend S2:

This 77-year-old male had a history of coronary artery disease (he had undergone proximal PCI of the first marginal branch of the RCX 12 years previously). He experienced angina during exercise, but was asymptomatic at rest. The substrate (figure S2 A) was a complex subtotal bifurcation lesion of the proximal RCX (Medina 1/1/1). Additionally, he had a borderline lesion (70% stenosis) of the proximal RCA (not shown). When attempting to wire the first marginal branch (which was already stented proximally), this branch became completely occluded. All attempts to reopen this branch failed. The final PCI result with side branch occlusion is shown in figure S2 B. The biomarker time courses for this Type 4a AMI are shown in figure S2 C. All the criteria of Type 4a AMI are met: angina; development of new negative T waves in ECG leads I and aVL; an angiographically documented complication (persistent side-branch occlusion); and a hs-cTnT increase >5-times the URL (14 ng/L). The increased baseline hs-cTnT concentration is most likely due to repeated myocardial ischemia with angina during physical exercise.

Abbreviations: percutaneous coronary intervention (PCI), circumflex coronary artery (RCX), right coronary artery (RCA), electrocardiogram (ECG), acute myocardial infarction (AMI), high-sensitivity troponin T (hs-cTnT)

Supplemental figure S3: Interpretation of cardiac troponin T in a 39-year-old male amateur cyclist who had to abandon a marathon cycling event after approximately 20 hours

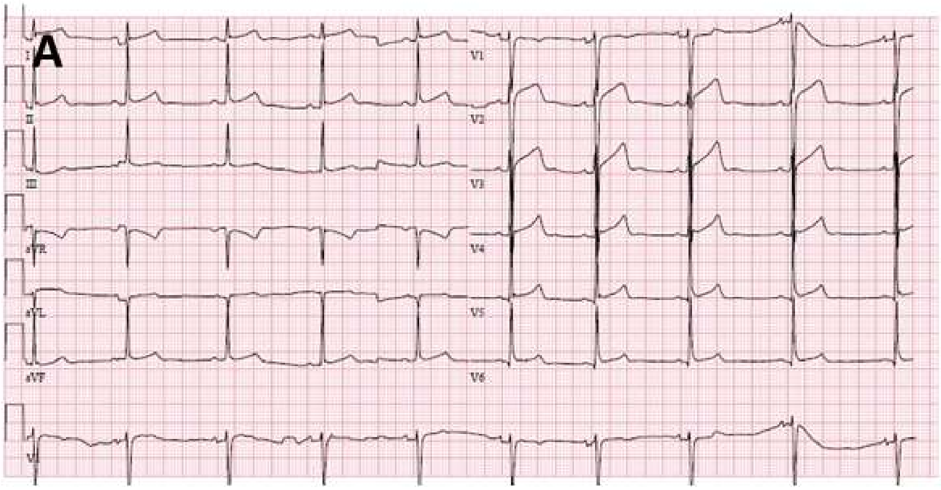

B	hs-cTnT (ng/L)	CK (U(L)	Leucocytes (G/L)	CRP (mg/L)
**Admission**	19.0	776	9.8	27.3
**1 h post**	18.5	703		
**18 h post**	14.1	350	5.3	13
**42 h post**	7.7	212	5.4	4

Figure legend S3:

This patient was admitted to the emergency department with the key symptoms of worsening dyspnoea on exertion and angina depending on body position and breathing. His ECG showed signs of early repolarisation (figure S3 A) without change in serial recordings. The chest X-ray performed on admission showed no pathologies, particularly no signs of pneumonia. His key laboratory parameters are listed in figure S2 B. A coronary computed tomography angiography revealed a calcium score of 0 with normal coronary arteries and no arteriosclerotic plaques or coronary anomalies. As is typical for long-term endurance sportsman, the left ventricular stroke volume was increased and the left ventricle was slightly dilated, and the left ventricular ejection fraction and right ventricular function were normal. These findings were confirmed by echocardiography. Before the start of the competition, he was fit and showed no signs of infection. On admission he reported a cough, but the physical examination, including oxygenation and body temperature, was normal. The hs-cTnT concentration was slightly increased on admission, falling steadily, showing a return within the URL before discharge. The maximum concentration was found on admission and was <3-times the URL, which is consistent with an exercise-induced cTn release. High creatine kinase activities are to be expected with such a high skeletal muscle workload. However, due to his advanced age, coronary computed tomography angiography was performed to rule out coronary artery disease. C-reactive protein was slightly increased on admission, consistent with a heavy endurance exercise workload, and returned to normal limits by discharge. Leucocyte count was within the reference range, but a differential blood count the morning after admission revealed relative lymphocytosis of 71%. The patient wanted to be discharged because he was visiting from abroad for the competition. The discharge diagnosis was suspicion of a viral respiratory infection. Although acute myocarditis was unlikely, cardiac magnetic resonance imaging to rule it out definitively was not possible before discharge. This imaging modality was recommended in case of persistent symptoms.

Abbreviations: high-sensitivity troponin T (hs-cTnT), creatine kinase (CK), C-reactive protein (CRP), upper reference limit (URL), electrocardiogram (ECG)

Supplemental figure S4: Time course of cardiac troponin T concentrations in a patient who underwent successful primary percutaneous coronary intervention of a dominant right coronary artery

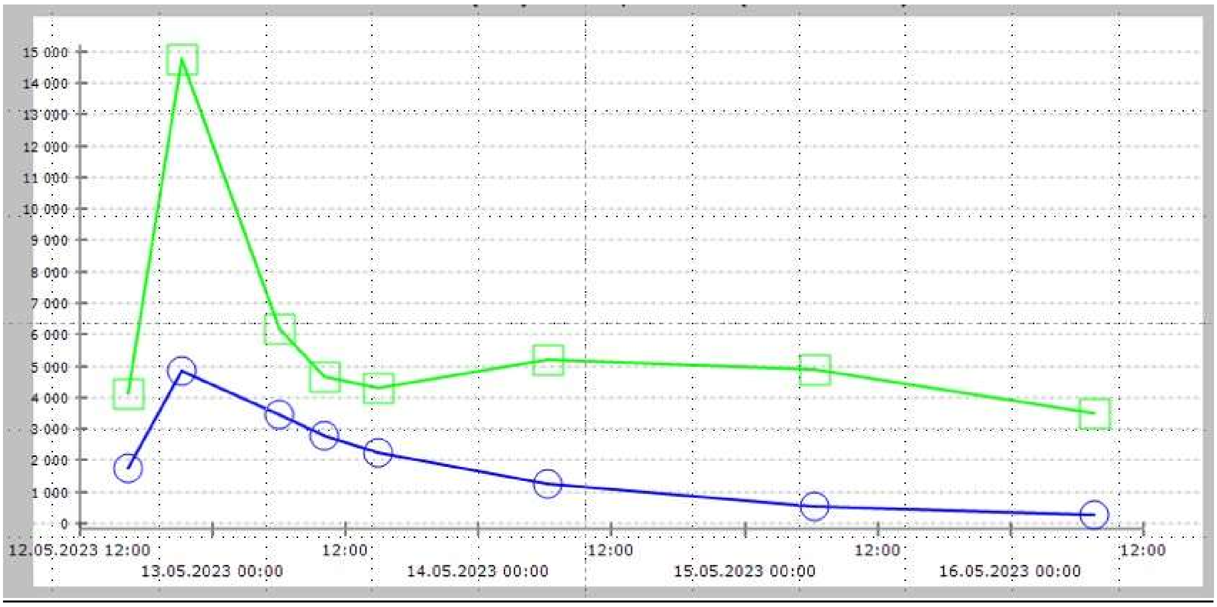

High-sensitivity cardiac troponin T (hs-cTnT) is plotted in green as ng/L and creatine kinase (CK) activities are plotted in blue as U/L.

The typical biphasic release pattern of cardiac troponin T is shown. In this patient, successful reopening of the infarct-related coronary artery and complete reperfusion of the infarcted myocardium resulted in a rapid high hs-cTnT peak about 12 hours after symptom onset, followed by a rapid decline (washout phenomenon). The second, later peak was much lower. CK showed the typical pattern of early reperfusion with an early peak as well.

Supplemental figure S5: Time courses of cardiac biomarkers in a 45-year-old male patient with ST-segment elevation acute myocardial infarction (STEMI)

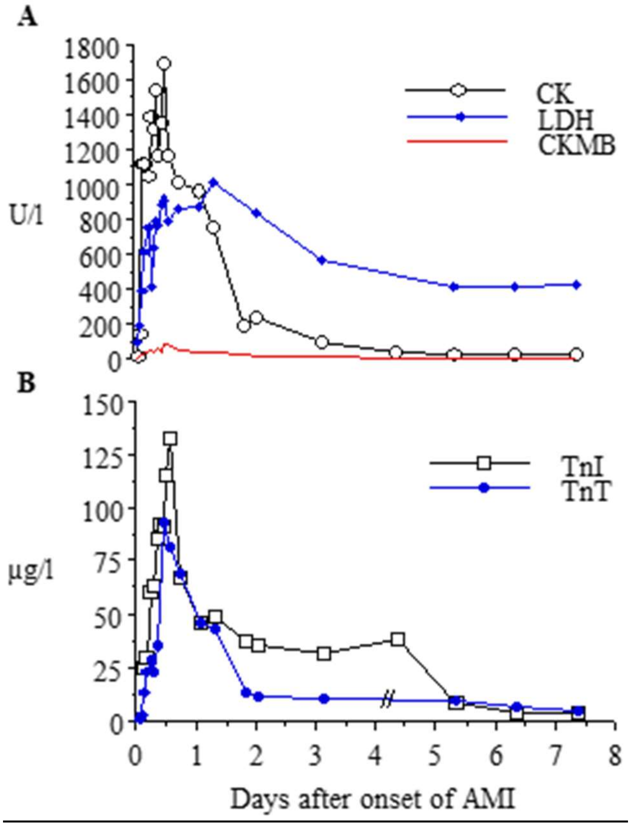

The activity time courses of cardiac enzymes are shown in figure S5 A and the time courses of cardiac troponins are shown in figure S5 B. Cardiac troponins were measured using previous, less sensitive assays and are therefore given in µg/L. A biphasic release pattern is evident for cTnI. Due to insufficient sample volume, cTnT could not be tested on day 4 and no concentration is available for this time point.

<u>Abbreviations:</u> creatine kinase (CK), lactate dehydrogenase (LDH), creatine kinase isoenzyme MB (CKMB), cardiac troponin I (cTnI), cardiac troponin T (cTnT)

(Adapted from Mair, J.; Puschendorf, B.; Michel, G. Clinical significance of cardiac contractile proteins for the diagnosis of myocardial injury. *Advan. Clin. Chem.* **1994**, 31, 63-98.)

Supplemental figure S6: Time course of cardiac troponin T concentrations in a patient with inferior wall ST-segment elevation acute myocardial infarction and no reflow after primary percutaneous coronary intervention

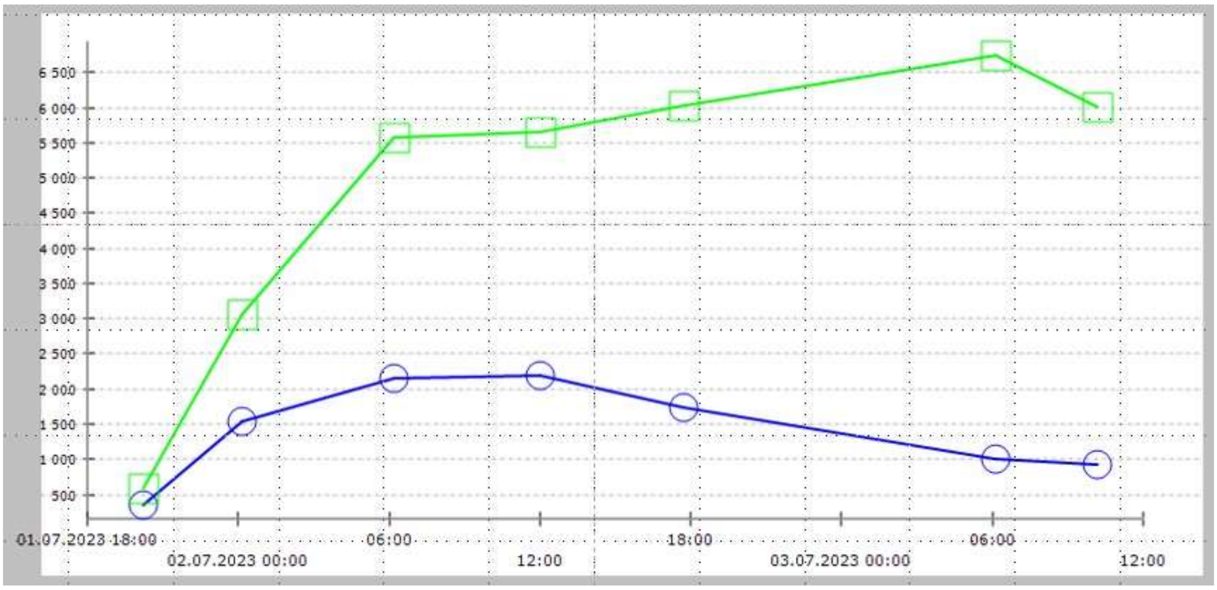

High-sensitivity cardiac troponin T (hs-cTnT) is plotted in green as ng/L and creatine kinase (CK) activities are plotted in blue as U/L.

The typical biphasic release pattern of cardiac troponin T is shown. In this patient, who experienced no reflow phenomenon in the infarct-related coronary artery and the infarcted myocardium (with only treatment-resistant, delayed TIMI 1 blood flow after stent implantation), a small initial hs-cTnT peak occurred approximately 24 hours after symptom onset, followed by a delayed maximum. CK also exhibited the characteristic pattern of failed reperfusion, reaching a maximum approximately 24 hours after symptom onset.

Supplemental figure S7: Cardiac biomarker testing results from 2 recent visits of a 19-year-old male suffering from Becker's muscular dystrophy

	Creatine kinase (U/L)	hs-cTnT (ng/L) URL <14	hs-cTnI (ng/L) URL <34	NT-proBNP (ng/L)
Baseline	6277	41.6	18.9	<50
2-year follow-up	1996	32.8	19.7	<50

His father also suffered from this disease. Genetic testing confirmed the diagnosis by demonstrating a mutation of the dystrophin gene (a deletion of exons 45-47). Throughout his whole life, this patient had no cardiac symptoms and a normal exercise capacity. ECGs and echocardiograms performed at both visits were normal. NT-proBNP concentrations were normal as well. Cardiac magnetic resonance imaging performed at the last visit was normal too. Discordant hs-cTnT and hs-cTnI results are often seen in patients with chronic skeletal muscle dystrophies, which is consistent with the possibility of re-expression of the cTnT gene in chronically injured skeletal muscle. It should be noted that, although frequent, cardiomyopathy in patients with Becker's disease usually does not start to develop before the third decade of life.

Abbreviations: high-sensitivity troponin T (hs-cTnT), high-sensitivity cardiac troponin I (hs-cTnI), N-terminal B-type natriuretic peptide (NT-proBNP), upper reference limit (URL), electrocardiogram (ECG), B-type natriuretic peptide (BNP)
